# Autonomous oscillation/separation of cell density artificially induced by optical interlink feedback as designed interaction between two isolated microalgae chips

**DOI:** 10.1038/srep24602

**Published:** 2016-04-21

**Authors:** Kazunari Ozasa, June Won, Simon Song, Mizuo Maeda

**Affiliations:** 1 Bioengineering Lab., RIKEN, 2-1 Hirosawa, Wako, Saitama 351-0198, Japan; 2 Department of Mechanical Convergence Engineering, Hanyang University, 222 Wangsipriro, Seongdong-gu, Seoul, 133-791, Korea; 3 Institute of Nano Science and Technology, Hanyang University, 222 Wangsipriro, Seongdong-gu, Seoul, 133-791, Korea

## Abstract

We demonstrate a designed interaction between two isolated cell populations of *Euglena gracilis* and *Chlamydomonas reinhardtii*, separately confined in two 25-square micro-aquariums of lab-on-chip size. The interaction was realized by interlinking two identical optical feedback systems, which measured the cell distribution. To analyze the cell populations, we measured the cell distribution in the 25 squares and irradiated the cells with a blue light pattern as an external stimulus. The cell distribution dataset was exchanged between the two systems. Governed by a designed interaction algorithm, the feedback systems produced a dynamic blue light illumination pattern that evoked the photophobic responses of both species. We also induced autonomous cell density oscillation and cell distribution separation and clustering, and analyzed how the types and diversities of the photophobic responses affected the oscillation period and separation and clustering. We conclude that artificial interlink feedback is a promising method for investigating diverse cell–cell interactions in ecological communities, and for developing soft-computing applications with living cells.

The spontaneous emergence of spatio-temporal rhythms and patterns is commonly observed in various natural ecological systems, in which multiple species of animals, plants, or microorganisms interact through environmental factors^1^,^2^,^3^,^4^. The autonomous generation and temporal evolution of rhythmic oscillations and pattern transitions in a micro-ecological systems has attracted much interest in software and optimization research^5^,^6^,^7^,^8^,^9^. The cells in microscopic ecological systems have developed survival strategies against their harsh environments, leading to sophisticated behaviors such as exploration, adaptation, competition^10^,^11^, and symbiosis^12^,^13^. Such sophisticated behaviors in nature have inspired computational algorithms for dynamic optimization problems, such as flow network optimization^14^ and effective path planning^15^. To reproduce the autonomous rhythmic oscillations and pattern transitions of microorganisms in nature, a laboratory-scale system is needed in which we can control and observe living cells.

However, an experimental system with well-controlled cell–cell interactions is difficult to construct, since the type, intensity, speed, and spatial scale of the interaction are determined by nature, which is beyond human control. Numerical models offer an alternative^16^,^17^,^18^, but cannot adequately capture the cell–cell variations, adaptability, and other characteristics of real cell communities. Consequently, numerical analysis yields simple simulation results rather than the diverse behaviors of nature. One possible solution is to develop an experimental model system with living microorganisms. In such a system, cell–cell interactions could be triggered by artificial stimuli that evoke phototactic^19^,^20^ or chemotactic^21^,^22^ responses of the cells.

Here we demonstrate an artificial cell–cell interaction between two species of motile photo-responsive microalgae, *Euglena gracilis* and *Chlamydomonas reinhardtii*, using two sets of an optical feedback system with a lab-on-chip scale culture dish. The distributions of the microalgae in separated culture chips were converted to numerical datasets, and exchanged from one system to the other. From the exchanged datasets, temporal illumination patterns were produced in each system according to an interlinking algorithm. Iterative irradiation of these patterns on each microchip induced the photophobic responses of the contained microalgae. The activity and distribution of the cells in both isolated chips was correlated by the interlink feedback and evolved autonomously. A pattern-fixed and pattern-variable illumination algorithm generated autonomous rhythmic oscillations and pattern transitions, respectively, in the cell density distribution. The photophobic responses of *E. gracilis* and *C. reinhardtii* were characterized by analyzing the autonomous oscillation period and domain clustering in the cell density distribution. The potential contributions of the developed system to ecological community research and soft computing are also discussed.

## Results

### Autonomous Cell-Density Oscillation

**Figure 1:**
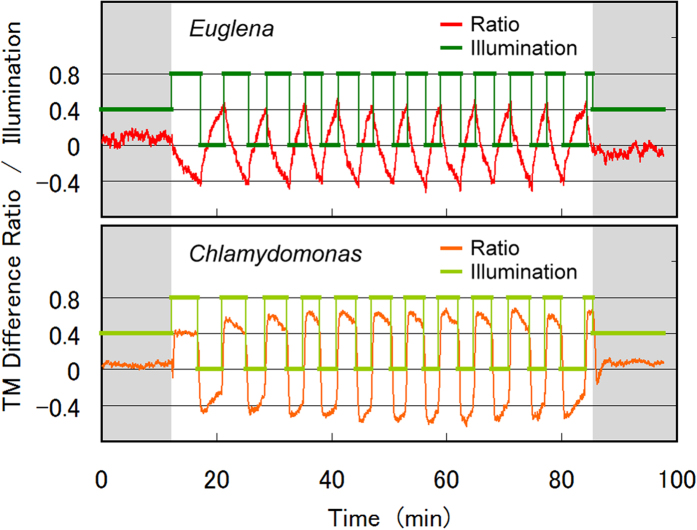
Autonomous oscillation generated by a two-state flipping algorithm with a prefixed threshold ratio of 0.4. Plotted are the illumination status and the (*TM*_*I*_ − *TM*_*II*_)/(*TM*_*I*_ + *TM*_*II*_) ratio (upper level = dish A; lower level = dish B). The feedback operation was active between time steps 500 (12.2 min) and 3,500 (85.5 min).

A typical oscillation evoked by Algorithms (1) and (2) of the interlink feedback is presented in Fig. 1. The threshold (*TM*_*I*_ − *TM*_*II*_)/(*TM*_*I*_ + *TM*_*II*_) ratio was set to 0.40. Once the feedback began operating at 12.2 min (time step 500), the *C. reinhardtii* cells in the illuminated squares (group I) were rapidly activated, and (*TM*_*I*_ − *TM*_*II*_)/(*TM*_*I*_ + *TM*_*II*_) increased in dish B (supplementary information: video movie AutoOsc2.mov). On the contrary, the *E. gracilis* cells in the illuminated squares gradually escaped to the non-illuminated squares, and the (*TM*_*I*_ − *TM*_*II*_)/(*TM*_*I*_ + *TM*_*II*_) slowly decreased in dish A, reaching the −0.40 threshold at 16.7 min. At this point, the illumination in dish B was flipped from group I to group II squares. Under the new illumination scheme, the *C. reinhardtii* cells in the group-II squares were quickly activated while those occupying group-I squares gradually resumed resting. In dish B, the ratio (*TM*_*I*_ − *TM*_*II*_)/(*TM*_*I*_ + *TM*_*II*_) reached the −0.40 threshold at 17.2 min, and the illumination was flipped in dish A. Sustained flipping of the illumination by the interlink feedback generated an autonomous oscillation with a period of 6.5 min. The oscillation was not completely uniform, but fluctuated as shown in Fig. 1. Such fluctuations are commonly observed in nature, reflecting the diverse and variable responses of living cells, and typify multi-layered complex systems. When the interlink feedback was terminated at 85.5 min (time step 3,500), the ratio (*TM*_*I*_ − *TM*_*II*_)/(*TM*_*I*_ + *TM*_*II*_) rapidly settled to zero.

**Figure 2:**
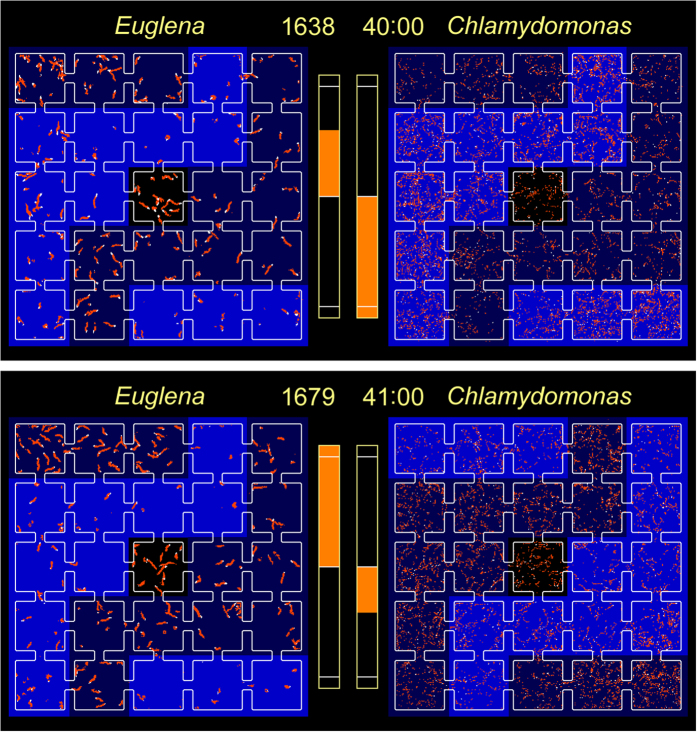
(a, top) Swimming traces in dishes A and B (occupied by *E. gracilis* and *C. reinhardtii* cells, respectively) at 40.0 min (time step 1,638). Illuminated group-II squares in both dishes are indicated in blue. The bar graphs show the (*TM*_*I*_ − *TM*_*II*_)/(*TM*_*I*_ + *TM*_*II*_) ratios. The white lines indicate the prefixed threshold ratio (0.4). The (*TM*_*I*_ − *TM*_*II*_)/(*TM*_*I*_ + *TM*_*II*_) ratio in dish A is 0.24, increasing gradually. The ratio in dish B has already exceeded −0.4. (b, bottom) Swimming trace image at 41.0 min (time step 1,679). The ratio (*TM*_*I*_ − *TM*_*II*_)/(*TM*_*I*_ + *TM*_*II*_) of dish A exceeds the threshold ratio 0.4, and the illumination flips to the group-I squares of dish B. The ratio in dish B is −0.17, increasing quickly.

Panels (a,b) of Fig. 2 show representative images of the micro-aquariums at 40.0 and 41.0 min, respectively, superimposed by the cells’ swimming traces and the illumination areas. At 40.0 min, the group-II squares in both dishes were illuminated. Although the *E. gracilis* cells in dish A were escaping from the group-I squares to group-II squares, the ratio (*TM*_*I*_ − *TM*_*II*_)/(*TM*_*I*_ + *TM*_*II*_) was 0.24 (below the threshold). The *C. reinhardtii* cells in the group-II squares of dish B were already activated, and those occupying group-I squares were gradually settling into the resting state. The ratio (*TM*_*I*_ − *TM*_*II*_)/(*TM*_*I*_ + *TM*_*II*_) of dish B exceeded the threshold after 38.3 min (−0.45 at 40.0 min). In dish A, the threshold was exceeded at 40.8 min, and the illumination in dish B was flipped. At 41.0 min, the *E. gracilis* cells in dish A were escaping further from the group-I squares, and (*TM*_*I*_ − *TM*_*II*_)/(*TM*_*I*_ + *TM*_*II*_) increased to 0.49. At 41.0 min, the *C. reinhardtii* cells in the group-II squares were still active while their counterparts in the group-I squares were partially quiescent. Consequently, (*TM*_*I*_ − *TM*_*II*_)/(*TM*_*I*_ + *TM*_*II*_) was small (−0.17).

As evident in Fig. 1, the *TM* changed much more slowly in dish A than in dish B. Thus, the oscillation period was predominantly determined by the *TM* change in dish A. Although the photophobic responses of *E. gracilis* are instantly evoked under blue light irradiation, the response action (tumbling) is not direct evacuation from the illuminated squares. Therefore, the response time of the *TM* change is dictated by the time required to escape the illuminated squares by accidental drift during tumbling. On the other hand, the photo-induced activation of *C. reinhardtii* directly initiates a *TM* change, which explains the faster *TM* response shown in Fig. 1. The fast *TM* change in dish B synchronizes the illumination of dishes A and B, with a phase delay of 0.43 min (Fig. 1).

**Figure 3:**
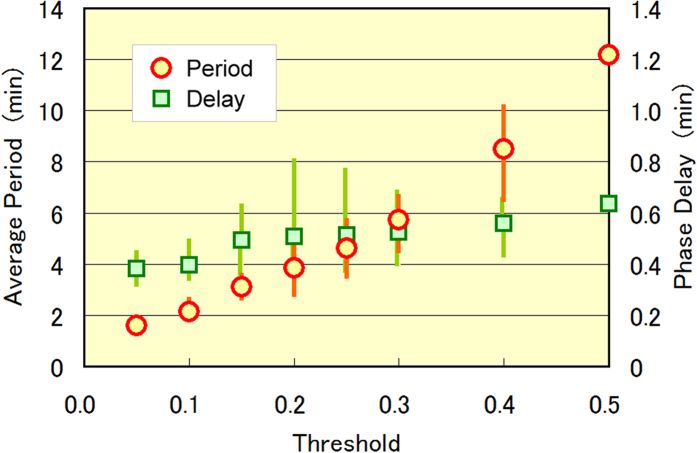
Dependence of oscillation period and phase delay on the prefixed threshold ratio. Results were obtained by the two-state flipping algorithm. Marks and bars indicate the average and spreading of the data in two or three experiments, respectively. The data at threshold ratio 0.5 are included for reference, since oscillations were observed in only one experiment. We speculate that the data are scattered by the circadian rhythms of the cells.

Figure 3 shows how the oscillation period and phase delay between dish A and B depend on the prefixed threshold ratio. As the threshold was increased from 0.05 to 0.5, the oscillation period increased almost linearly from 1.6 to 12 min, while the phase delay increased more slowly from 0.38 to 0.63 min. The oscillation period was governed by the escape speed of *E. gracilis* cells from the illuminated squares, which can be controlled by the size of the squares and the width of the connecting paths, as well as the selection of the 12 squares comprising the groups. In contrast, the phase delay was determined by the activation/resting responses of *C. reinhardtii* in each square, which are independent of micro-aquarium shape and group configuration. At thresholds of 0.45 or higher, the oscillations were less stable because the ratio (*TM*_*I*_ − *TM*_*II*_)/(*TM*_*I*_ + *TM*_*II*_) in dish B saturated at around 0.45, as seen in Fig. 1.

The algorithm of the above autonomous oscillation is the simplest two-sate flipping scheme with the fixed illumination patterns. By modifying the feedback algorithm and micro-aquarium configuration, the same combination of *E. gracilis* and *C. reinhardtii* will realize various autonomous oscillations or regulations, such as settling/oscillation bifurcation^23^, multi-state oscillations^24^, or active/refractory transitions^25^. Importantly, our scheme allows purposefully designed interaction algorithms, despite that the temporal evolution of the cell density in illuminated areas is governed by the complex survival strategies of two microalgae. Although rules (1) and (2) are deterministic, they may not precisely predict the complicated photophobic responses of the microalgae species, which are affected by stochastic processes such as cell cycles, circadian rhythms, metabolic statuses, and environmental factors. Furthermore, the responses are inhomogenous even among a homogeneous cell group, reflecting the cell–cell variations.

### Autonomous Separation and Clustering in High Cell-Density Areas

**Figure 4:**
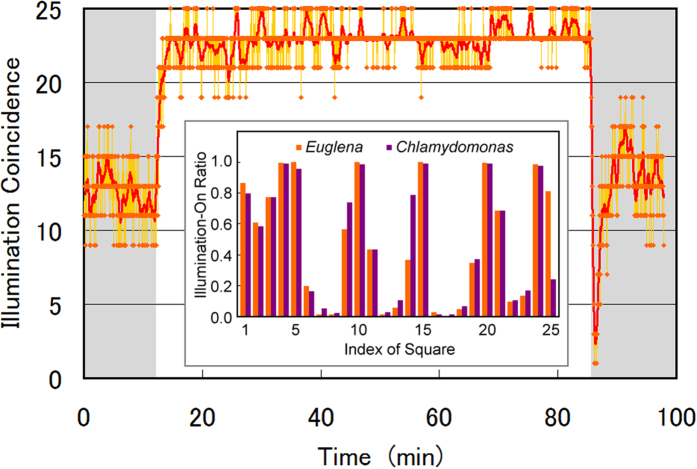
Temporal evolution of illumination coincidence between dishes A and B, defined as the number of corresponding squares in dishes A and B with the same illumination/non-illumination status. Red line indicates the moving average over ±10 time steps. The feedback operation was active from time steps 500 (12.2 min) to 3,500 (85.5 min). The inset is a histogram of the ratio (illumination-On time steps)/(total feedback time steps) in each square, indexed from upper left (1) to lower right (25). Except for Squares 9, 14, and 25, the ratios were mostly identical in dishes A and B, suggesting similar illumination/non-illumination of the corresponding squares in the two dishes.

Feedback algorithms (3) and (4) autonomously separate the high cell-density areas in a given micro-aquarium and cell population. Figure 4 shows the temporal evolution of illumination coincidence between the two dishes, i.e., the number of squares in dishes A and B with the same illumination/non-illumination status. Once the feedback operation started at 12.2 min, the illumination coincidence rapidly increased to 23 ± 2, indicating an almost identical illumination pattern in both dishes. The coincidence remained high at 23 ± 2 until the feedback was terminated at 85.6 min. The illumination coincidence indicates that corresponding squares in dish A and dish B have a larger and smaller *TM*, respectively. Therefore, the high illumination coincidence indicates that the spatial distributions of active cells are reversed in dishes A and B. In other words, this interlink feedback realized artificial separation of high cell-density areas between *E. gracilis* and *C. reinhardtii*.

**Figure 5:**
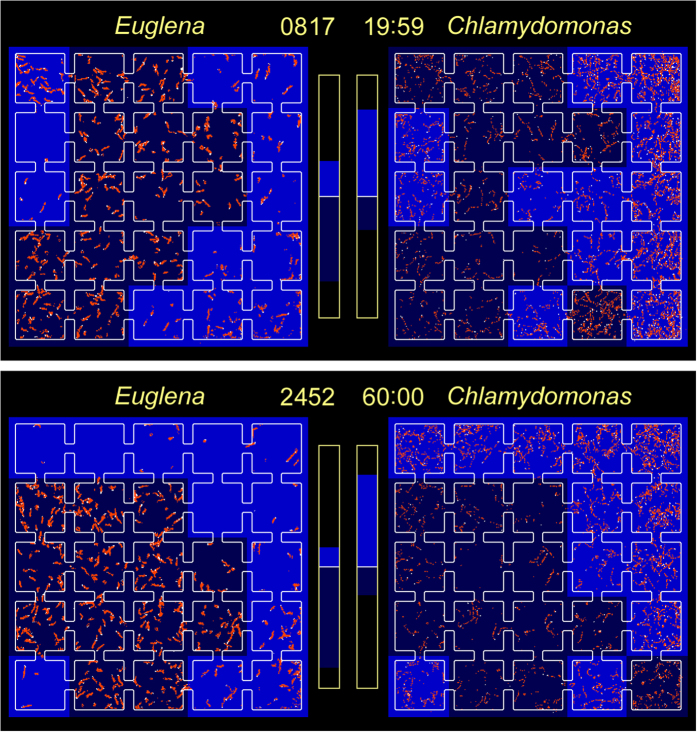
(a, top) Swimming traces of dish A (containing *E. gracilis*) and dish B (containing *C. reinhardtii*) at 20.0 min (time step 817). Upper and lower bar graphs show the *TM* ratio of illuminated squares and non-illuminated squares, respectively. The *TM* of the illuminated squares in dishes A and B was 28% and 72%, respectively. (b, bottom) Swimming trace image at 60.0 min (time step 2,452). The *TM* of the illuminated squares in dishes A and B was 24% and 84%, respectively.

The illumination pattern dynamically altered during the feedback operation. Panels (a) and (b) of Fig. 5 are transient images of the illumination patterns and swimming traces of the cells at 20.0 min and 60.0 min, respectively. At 20 min, the non-illuminated squares in dish A were already clustering into one domain, whereas the illuminated squares in dish B were still partially dispersed. Since *E. gracilis* more actively migrates from illuminated to non-illuminated squares than *C. reinhardtii*, domain formation is dominated by the photophobic responses of *E. gracilis*. The incomplete clustering of the illuminated squares in dish B suggests that some of the *E. gracilis* cells were still swimming in those squares. At 20 min, the *TM* of the non-illuminated squares in dish A and dish B comprised 72% and 71% of the total *TM*, respectively. At 60 min, the non-illuminated (illuminated) squares in dish A (B) were clustering more tightly than at 20 min (forming square-like rather than rectangular configurations). At this time, the *TM* of the non-illuminated (illuminated) squares in dish A (B) was 76% (84%). The spatial distributions of the *E. gracilis* and *C. reinhardtii* cells are almost inverted in Fig. 5(b).

The ratio (illumination-On time steps)/(total feedback time steps) in each square is plotted as a histogram in the inset of Fig. 4. The ratio approximates unity in squares indexed 4, 5, 10, 15, 20, and 24, revealing that those squares were almost constantly illuminated in both dishes. On the contrary, squares with ratios below 0.2 were usually non-illuminated throughout the experiment. The ratio in squares indexed 9, 14, and 25 largely differs between dishes A and B, suggesting that both *E. gracilis* and *C. reinhardtii*, were highly active or inactive in those squares.

**Figure 6:**
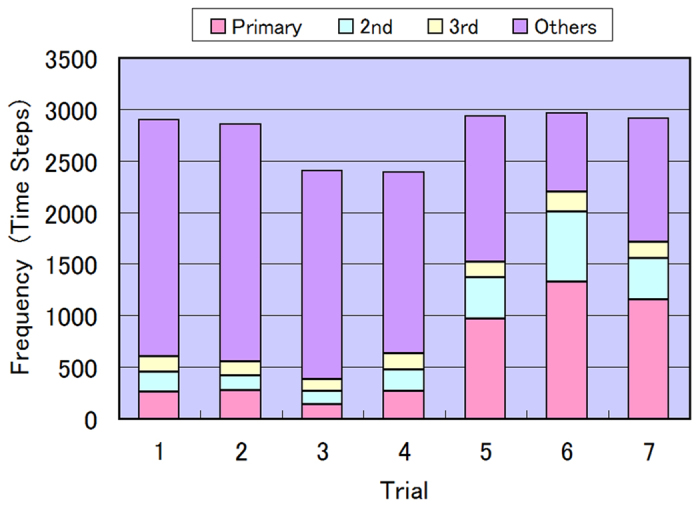
Appearance frequency of the clustering patterns exhibiting high reversal between dishes A and B in seven experiments. The histogram shows the number of time steps in which the illumination coincided in 21–25 squares during the feedback operation (3,000 time steps), sectioned into 1st, 2nd, and 3rd most frequent patterns, and other patterns. Maximum (minimum) number of high reversal clustering patterns was 279 (30), observed in Trial #3 (#6).

Figure 6 plots the appearance frequency of domain patterns exhibiting high reversal between dishes A and B, observed in seven experiments. Shown is the number of time steps for which the illumination coincidence was 23 ± 2 squares during the feedback operation (3,000 time steps). The results are binned into 1st, 2nd, and 3rd most frequent patterns, and other patterns. In each experiment, illumination coincidence was 80% or higher during the feedback operation. Among the seven experiments, coincidence in the 1st, 2nd, and 3rd most frequent patterns ranged from 16% to 74%. Relatively smaller frequencies in the top three patterns suggest that the various patterns of high illumination coincidence were in frequent dynamic transition. In most of the experiments, the non-illuminated squares gradually aggregated into one tightly clustered domain. Such clustering is attributable to the movements of the *E. gracilis* cells, which accidentally enter an illuminated square from the adjacent non-illuminated square. This activity induces on–off flipping of the illumination. If illumination flipping occurs with high probability, the number of boundaries between the illuminated and non-illuminated squares tends to reduce, promoting a single clustered domain of non-illuminated squares.

## Discussion

The oscillation periods and cluster patterns in the above results were determined not only by the feedback algorithms, but also by the intrinsic nature of the microalgae species; namely, by their diverse photo-responses (cell–cell variations), cell cycles, metabolic statuses, and adaptations to illumination. Diversity in the cells’ photophobic responses introduces fluctuations in the period of autonomous cell density oscillation, and promotes the clustering of high cell-density domains. Being closely related to photophobic response and motility, cell cycles and circadian rhythms also influence the interlink feedback results in a systematic manner, evoking gradual increase or decrease in the oscillation period and clustering speed. Because the oscillation period gradually decreased as the experiments were repeated, we speculate that the scattered data observed in the oscillation period (Fig. 3) and the top-three pattern frequencies (Fig. 6) were sourced from cell motility changes governed by the circadian rhythm. Meanwhile, the long-term evolution of the autonomous oscillation or separation of cell density depends on the survival strategies of the microalgae cells, including their adaptation to external stimuli, robustness against environmental change, and diversity of cell characteristics.

Our major interests are to establish a bio-system in which living cells interact according to their biological nature through external stimuli artificially provided in a programmable manner. We have demonstrated one example of such a bio-system; an interlink feedback system with a micro-aquarium of lab-on-chip size, where the cell density oscillated and the cell distribution became separated or clustered by interactions between the two micro-aquaria. Our experiment could be criticized as simply simulating the outcome, without identifying the cause, of such oscillations and separations. This criticism is valid if the cells exhibited a static response. However, the cells showed dynamic changes in their photophobic responses, such as sensitization and adaptation. Therefore, the observed oscillations and separations were not merely simulated by computer models, but were driven by the inherent nature of the individual cells. Nonetheless, our simple experimental algorithms generated complicated and unpredictable temporal evolutions of the oscillations and domain clustering, owing to the photophobic responses of the microalgae species. We reemphasize that a temporal evolution system in which natural living cells are driven by a PC-controlled feedback algorithm is a novel and promising concept for investigating cell–cell interaction mechanisms and phenomena in general.

In natural ecological communities, diverse cell–cell interactions are mediated by environmental factors, such as chemical substances released from other cells. Attractive or repellant pheromones are the typical example. As demonstrated in the cell-distribution separation and clustering experiment, our artificial interlink system mimics the cell–cell interactions induced by environmental factors, and reproduces the temporal evolution of ecological communities. Moreover, we can control and dynamically alter the environmental effects, which cannot be accomplished in natural ecological communities. Thus, our interlink feedback scheme will expand existing researches on the temporal evolution of ecological communities.

Our interlink feedback system is potentially applicable to soft-computing inspired by living cells. We have already investigated the photophobic responses of *E. gracilis* in a 16-branch micro-aquarium under a single-feedback mechanism, and incorporated those responses into a neural network model^26^,^27^. In that demonstration, two favorable characteristics of microalgae-based neurocomputing were elucidated for a simple combinatorial optimization problem: (1) obtaining a best solution to the problem and (2) searching for multiple solutions via dynamic transitions among the best solutions^27^,^28^. More flexibility and higher performance in microalgae-based neurocomputing is expected when two or more systems containing different micralgae species are cooperatively interlinked. For instance, under different collaborative algorithms, one system can seek an overall optimization while the other finds the details, or one system can exploit a better solution far from the current solution while the other searches nearby solutions.

## Conclusion

We demonstrated optical interlink feedback between two isolated populations of *E. gracilis* and *C. reinhardtii* cells, with confining the cells within 25-square micro-aquaria of lab-on-chip size. To provide the interlink feedback through the photophobic responses of the cells, we irradiated blue light patterns generated by a customized algorithm. The interlink algorithm of the simplest two-sate flipping scheme with the fixed illumination patterns generated autonomous cell-density oscillations in the fixed area in the both dishes. By another algorithm, we irradiated blue light on the one-half of squares exhibiting higher cell activities in the counter-dish, and achieved autonomous separation and clustering of high cell-density areas, i.e., reversed cell distribution patterns in the target and counter-target dishes. Using this optical interlink feedback scheme, we can design bio-systems in which living cells interact through an artificially designable algorithm, and investigate the nature of living cells by autonomously time-evolving their activities in artificially interlinked ecological communities. The interlink feedback system is also applicable to soft computing inspired by living cells.

## Methods

### Cells in Micro-Aquarium

As test species, we selected the microalgae *Euglena gracilis* (Z-strain)^29^,^30^,^31^,^32^ and *Chlamydomonas reinhardtii* (CC-125 strain)^33^,^34^,^35^,^36^ for their motility, photo-responsiveness, and easily observed body sizes and swimming speeds. The typical body size and swimming speed of *E. gracilis* is 20–100 μm and 30–90 μm/s, respectively; the corresponding values for *C. reinhardtii* are 10–30 μm and 20–100 μm/s, respectively. The cells were maintained in Cramer–Myers’ (CM) medium^37^ as suspensions in microtubes, under a weak room light for photosynthesis. Both species are responsive to blue light but exhibit different photophobic responses (as shown in subsection 2.3).

**Figure 7:**
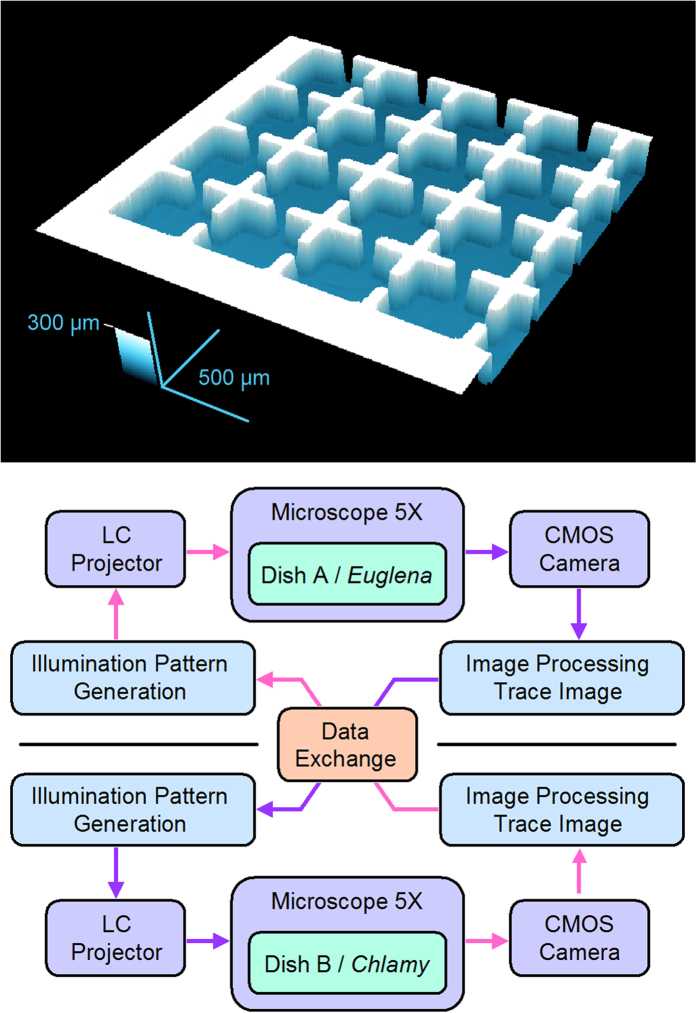
(a, top) Image of 25-square micro-aquarium observed under a confocal microscope (only 16 of the 25 squares are visible). Each square is 480 μm wide and 120 μm deep. The chip is made of PDMS, and covered with a glass slip after the introduction of cells. (b, bottom) Diagram of signal flow in two optical feedback systems interlinked by data exchange. The dataset of 25 *TM*s, calculated by image-processing the trace images of the micro-aquarium, is transferred to the counter system. The exchanged *TM* dataset is input to a customized algorithm that generates an illumination pattern in each system, which is then projected to the micro-aquarium. Blue-light illumination induces photophobic responses in the *E. gracilis* and *C. reinhardtii* cells occupying separate micro-aquaria.

The cells were confined within a 25-square micro-aquarium (a closed chamber for swimming cells) made of polydimethylsiloxane (PDMS). The chamber layout is shown in Fig. 7(a). Dividing the chamber into 25 cells simplified the cell distribution analysis. Each square was 480 μm wide and 120 μm deep, sufficiently large to accommodate several tens of cells. The squares were interconnected by square passages (width = length = 90 μm). Droplets of cell suspension, one containing approximately 400 cells of *E. gracilis*, the other containing approximately 1300 cells of *C. reinhardtii*, were separately injected into two isolated micro-aquaria. The higher cell density of *C. reinhardtii* compensates for its much smaller swimming trace than *E. gracilis*. Each micro-aquarium was covered with a glass slip, and contained in a 25-mm diameter dish. To simplify the text, we hereafter refer to the dishes containing *E. gracilis* and *C. reinhardtii* as dish A and dish B respectively. Confined in the micro-aquarium, the *E. gracilis* cells continued swimming straightforward, changing their swimming direction at each wall, but many of the *C. reinhardtii* cells gradually stopped swimming. The resting *C. reinhardtii* cells were activated and resumed swimming under blue light illumination.

### Interlink Feedback System

Our designed optical feedback system both observes and optically stimulates the cells^38^. We prepared two feedback systems, one for each micro-aquarium, and observed the cell movements under an optical microscope (Olympus, BX51) with a 5X objective lens and video camera imaging (Trinity, IUC-200CK2). The images were processed on a PC (Fujitsu, MG/D70N), and converted to 25 values that we named *trace momentum* (*TM*), defined as the number of pixels covered by the swimming traces in each square^26^. Each *TM* represents the cell activity within the square; the higher the *TM*, the more cells swim at faster speeds. The 25 *TM* values acquired by each system were exchanged between the two PCs using a LAN connection for interlink feedback (Fig. 7(b))^39^.

The main advantage of our optical feedback system is that an arbitrary spatial pattern can be sequentially irradiated onto the micro-aquarium. Here, pattern irradiation was performed by a liquid-crystal (LC) projector (Panasonic, PT-VX400) connected to a data-processing PC and reduction lenses. The PC was programmed with an algorithm that dynamically generates (1024 × 768) pixel images. These images were emitted from the LC projector and converted by the reduction lenses to a (5.0 × 3.7) mm^2^ illumination pattern. The pattern size was adjusted to fit the micro-aquarium on the microscope stage. By this scheme, we can independently and dynamically illuminate each square of the micro-aquarium. The illumination was introduced from the bottom side of the micro-aquarium. The illumination pattern was produced from the 25 *TM*s transferred from the counter-system, and a separate algorithm was designed for each experiment (see subsection 2.3). In all experiments, the blue light intensity of the feedback illumination was fixed at approximately 20 mW/cm^2^, sufficient to evoke photophobic responses in both *E. gracilis* and *C. reinhardtii*. The basic performance of our single optical feedback system and the changing cell distribution of *E. gracilis* under photophobic stimulus have been presented in our previous reports^38^. Therein, we also discussed the application of our *E. gracilis* experiments to neural network soft-computing.

### Photophobic Responses and Feedback Algorithms

Under 20 mW/cm^2^ blue light illumination, *E. gracilis* and *C. reinhardtii* exhibit different photophobic responses. The *E. gracilis* cells transit from straightforward swimming to on-site tumbling^40^,^41^,^42^,^43^,^44^. When a square is illuminated with blue light, the exposed cells drift as they tumble, and accidentally escape to a non-illuminated square. Furthermore, cells approaching the illuminated square turn at the illumination border and thereby evade the square. Consequently, the number of cells in the illuminated square gradually decreases, and more cells occupy the non-illuminated squares. Therefore, the *TM* decreases in the illuminated squares and increases in the non-illuminated squares.

*C. reinhardtii* cells are awakened by the blue light and resume swimming. When a square is illuminated by blue light, the occupant cells escape by opposing the direction of the illumination^45^,^46^,^47^,^48^. However, since the blue light is irradiated from the bottom of the micro-aquarium, the cells fail to find the path to non-illuminated squares. Consequently, the number of swimming cells increases in the illuminated squares but remains more-or-less static in the non-illuminated squares. In this case, the *TM* increases in the illuminated squares and is largely unchanged in the non-illuminated squares.

Accounting for the different *TM* responses of *E. gracilis* and *C. reinhardtii* to blue light, we designed two feedback algorithms as described below. For the oscillation experiments, 25 squares in each dish were divided into two groups of 12 squares (groups I and II; see panels (a,b) of Fig. 2); the center square was always non-illuminated. The 12 squares in any one group were simultaneously illuminated, while the squares in the other group were simultaneously non-illuminated. The 12 *TM*s in group I (II) were summed as *TM*_*I*_ (*TM*_*II*_). The group to be illuminated at the next time step was decided by the following algorithm.

1. At the beginning of the interlink feedback, illuminate the squares in group I.
2. When the *TM* difference between groups I and II relative to the total *TM* of both groups i.e., (*TM*_*I*_ − *TM*_*II*_)/(*TM*_*I*_ + *TM*_*II*_), exceeds a prefixed threshold (0.05–0.40), illuminate the same group in the counter-dish in the next time step. The above rule (2) is a two-state flipping algorithm with a prefixed threshold, which flips the illumination state on or off according to the balance between the cell activity in the counter-dish and the pre-determined limit. For the cell-density separation and clustering experiments, the illumination of each square was governed by the following algorithm:
3. In the current time step, select the 12 lowest *TMs* in dish A; in the next time step, illuminate the corresponding squares in dish B.
4. In the current time step, select the 12 highest *TMs* in dish B; in the next time step, illuminate the corresponding squares in dish A.

The above rules (3) and (4) correspond to a ranking algorithm, which selects the 12 high and low scoring squares based on their cell activity in the counter-dish. The *C. reinhardtii* cells are photo-activated in the 12 squares, corresponding to those containing more *E. gracilis* cells than the remaining 12 squares in the counter dish. Meanwhile, the *E. gracilis* cells are repulsed by illumination from the 12 squares, corresponding to those containing more *C. reinhardtii* cells than the remaining 12 squares in the counter dish.

The illumination patterns were refreshed in each time step of the feedback cycle at a rate of 0.68 Hz (1.47 s/cycle). The refresh timing of each system was synchronized within an error of ±1 cycle. Each experiment was executed through 4,000 time steps, suspending the feedback illumination during the first and last 500 time steps. Experimental duration was 98 min.

## Additional Information

**How to cite this article**: Ozasa, K. *et al.* Autonomous oscillation/separation of cell density artificially induced by optical interlink feedback as designed interaction between two isolated microalgae chips. *Sci. Rep.* **6**, 24602; doi: 10.1038/srep24602 (2016).

## Supplementary Material

Supplementary Information

Supplementary Video
