# Supplementary material for: Autonomous oscillation/separation of cell density artificially induced by optical interlink feedback as designed interaction between two isolated microalgae chips
Source: Sci Rep. 2016 Apr 21;6:24602. doi: 10.1038/srep24602 (PMC4838927; doi:10.1038/srep24602)
Supplement: Supplementary Information [file srep24602-s1.pdf]

Scientific Reports

SREP-15-23408A

Autonomous oscillation/separation of cell density artificially induced by optical interlink feedback as designed interaction between two isolated microalgae chips

Kazunari Ozasa, June Won, Simon Song, Mizuo Maeda

Video legends

AutoOsc2.mov

Autonomous oscillation generated by a two-state flipping algorithm with a prefixed threshold ratio of 0.4. The video shows the swimming traces observed in dishes A and B, occupied by *E. gracilis* and *C. reinhardtii*, respectively. Once the feedback began operating at 12.2 min (time step 500), the *C. reinhardtii* cells in the illuminated squares (group I) were rapidly activated, and the deviation ratio $(TM_I - TM_{II})/(TM_I + TM_{II})$ increased in dish B. On the contrary, the *E. gracilis* cells in the illuminated squares gradually escaped to the non-illuminated squares, and the $(TM_I - TM_{II})/(TM_I + TM_{II})$ slowly decreased in dish A, reaching the -0.40 threshold at 16.7 min. At this point, the illumination in dish B was flipped from group I to group II squares. Under the new illumination scheme, the *C. reinhardtii* cells in the group-II squares were quickly activated while those occupying group-I squares gradually resumed resting. In dish B, the ratio $(TM_I - TM_{II})/(TM_I + TM_{II})$ reached the -0.40 threshold at 17.2 min, and the illumination was flipped in dish A. Sustained flipping of the illumination by the interlink feedback generated an autonomous oscillation with a period of 6.5 min.
